# Quantifying the Impact of Motions on Human Aiming Performance: Evidence from Eye Tracking and Bio-Signals

**DOI:** 10.3390/s24051518

**Published:** 2024-02-26

**Authors:** Yuzhang Li, Xinming Li, Peter R. Grant, Bin Zheng

**Affiliations:** 1 Department of Mechanical Engineering, University of Alberta, Edmonton, AB T6G 1H9, Canada; yuzhang.li@ualberta.ca (Y.L.);; 2 Institute for Aerospace Studies, University of Toronto, Toronto, ON M3H 5T6, Canada; peter.grant@utoronto.ca; 3 Department of Surgery, University of Alberta, Edmonton, AB T6G 2B7, Canada

**Keywords:** reciprocal aiming task, Fitts’s Law, in-motion conditions, movement control, eye tracking

## Abstract

Working on a moving platform can significantly impede human performance. Previous studies on moving vehicles have often focused on the overall impact on general task performance, whereas our study’s emphasis is on precise hand movements, exploring the interaction between body motion and the escalation of task difficulty. We recruited 28 participants to engage in reciprocal aiming tasks, following Paul Fitts’s setting, under both in-motion and stationary conditions. The task index of difficulty (ID) was manipulated by varying the width of the targets and the distance between the targets. We measured participants’ movement time (MT), performance errors, and monitored their eye movements using an eye-tracking device, heart rate (HR), and respiration rate (RR) during the tasks. The measured parameters were compared across two experimental conditions and three ID levels. Compared to the stationary conditions, the in-motion conditions degraded human aiming performance, resulting in significantly prolonged MT, increased errors, and longer durations of eye fixations and saccades. Furthermore, HR and RR increased under the in-motion conditions. Linear relationships between MT and ID exhibited steeper slopes under the in-motion conditions compared to the stationary conditions. This study builds a foundation for us to explore the control mechanisms of individuals working in dynamic and demanding environments, such as pilots in airplanes and paramedics in ambulances.

## 1. Introduction

Human motor skills are controlled by complex mechanisms involving the coordination of multiple nervous and sensory systems. Motor skills help humans maintain balance, perceive the environment, and provide precision manipulation to the targets [1,2]. In daily practice, people must execute motor tasks, especially precise aiming tasks, in various work environments. For example, surgeons have to perform intricate aiming tasks during laparoscopic surgeries [3,4,5,6], drivers have to aim at and touch different control buttons within moving vehicles [7,8,9], and pilots have to delicately aim at and touch sophisticated cockpit control panels in aircrafts [2,5,10].

Motor skills are critical for jet fighter pilots, as they help in manipulating the control stick, which is responsible for the maneuverability of the aircraft [2]. To precisely control the aircraft, pilots need to simultaneously process information from their visual, auditory, proprioceptive, tactile, and vestibular pathways; a small error in the sensorimotor pathway and motor skills could cause severe aircraft instability, potentially leading to fatal consequences. Studies have shown that pilots tend to show cognitive fatigue when performing more challenging tasks [11], leading to higher cognitive load [12,13], thus pilots are more likely make operational errors while performing challenging tasks [14]. In the healthcare sector, surgeons face significant challenges when executing delicate motor tasks such as aiming at objects and performing tasks like knotting and cutting threads [15]. These challenges become more pronounced when paramedics need to perform complex aiming tasks under in-motion conditions. Paramedics have significant difficulty performing effective lifesaving procedures when the ambulance’s speed is over a certain limit [16]. It takes an average of 8 miles (17.3 min) for patients to get to the emergency department [17]. Thus, it is essential to examine the impact of moving vehicles on paramedics’ performance [18], especially on aiming skills, to save more patients. Researchers have made a noteworthy discovery regarding the impact of microgravity effects in parabolic flight situations on motor skills when one is tasked with tying laparoscopic surgical knots on simulated skins. This unique flight condition resulted in a substantial deterioration in the quality of the knots tied and a significant increase in the force applied to the surgical instruments when compared to the controlled ground situation [5]. However, these studies did not systematically quantify task difficulties using measurable parameters, specifically measure performance in the aiming tasks with increasing task difficulties, or compare participants’ physiological signals.

Several studies have highlighted the impact of in-motion conditions on human performance. In a study by Dodd et al. [19], participants were subjected to simulated turbulence while performing touchscreen tasks. The results showed that turbulence led to longer task times, increased data entry errors, higher fatigue ratings, and reduced performance compared to non-turbulent conditions. Another study explored touchscreen usage in different positions within a flight simulator, revealing longer task times and increased error rates during turbulence, along with heightened workloads and arm fatigue [20]. Furthermore, another experiment was conducted to explore biodynamic feedthrough in a simulated vibratory environment. It revealed an increased susceptibility to touch errors during turbulence [21]. In an investigation that used a roller coaster to simulate cockpit motions for pilots, the participants performed various touchscreen tasks. The results showed that task completion time and interactions with objects significantly increased under in-motion conditions compared to stationary conditions [22]. Yet another study focused on the impact of vertical vehicle ride motion on reaching tasks within vehicles. In this study, the researchers simulated various vehicle motions and found that vertical movements negatively affected the accuracy of the subjects and their task completion times [23]. Another experiment’s results showed that a control display is superior to other cockpit interfaces, with higher input accuracy and less movement time [24]. However, these studies did not systematically quantify task difficulties using measurable parameters, specifically measure performance or eye metrics in the aiming tasks with increasing task difficulties, or compare participants’ physiological signals.

Accordingly, some touchscreen methods have been introduced to improve human performance, especially during simulated flight tasks. Human performance has been tested in turbulence and vibration environments. Nevertheless, a few research gaps can be identified: (1) the task difficulty levels are not clarified and quantified based on measurable parameters; (2) the understanding of the mechanisms and performances involved in pure aiming and touching tasks under in-motion conditions is limited; (3) controlled laboratory studies on the impact of lateral accelerations with jerks and comparisons of overall performance and physiological signals between stationary and in-motion conditions are scarce; (4) a clear relationship between MT-ID and Error-ID and their quantification have not been provided for in-motion conditions; (5) approaches for analyzing and comparing eye fixation and saccade metrics together with task performances under in-motion and stationary situations are rare.

To bridge these gaps, this study adopted Fitts’s reciprocal aiming paradigm [25] and Shannon–Hartley theorem [26] to investigate how humans adapt to the impact of motions on aiming motor skills. The Fitts’s reciprocal aiming paradigm stands out as one of the most effective methods to quantify aiming task performance under the influence of different task difficulties by altering target weights and distances. In this approach, we intricately integrated Fitts’s Law paradigm with in-motion platforms to measure aiming performance along with some bio-signal changes.

Specifically, this study introduced aiming tasks with varying task IDs in both stationary and in-motion conditions and compared MT, durations of eye fixations and saccades, errors, HR, and RR between the two conditions. Thus, this study aimed to quantify how the abovementioned parameters and ID are related to in-motion conditions. This study further analyzed and discussed the specific potential reasons behind the phenomena, which may involve different factors in human movement control pathway.

The hypotheses for this study were as follows: (a) compared to stationary conditions, MT and errors in the aiming task and participants’ mental and physical workloads will significantly increase during in-motion conditions; (b) MT and errors in the aiming task and participants’ mental and physical workloads will increase as the task ID increases; (c) the deterioration of performance between in-motion and stationary conditions will be more pronounced with the increasing of task IDs; (d) participants will spend significantly longer times on eye saccades and fixations to locate and lock on to the targets under in-motion conditions than under stationary conditions and when the task ID increases.

## 2. Materials and Methods

### 2.1. Setup and Apparatus

This study was conducted in the Occupational Ergonomics Research Lab at the University of Alberta (UofA). This study was reviewed and approved by the UofA Health Research Ethics Board. Informed consent was obtained from all participants involved in this study before the experiment.

**Figure 1:**
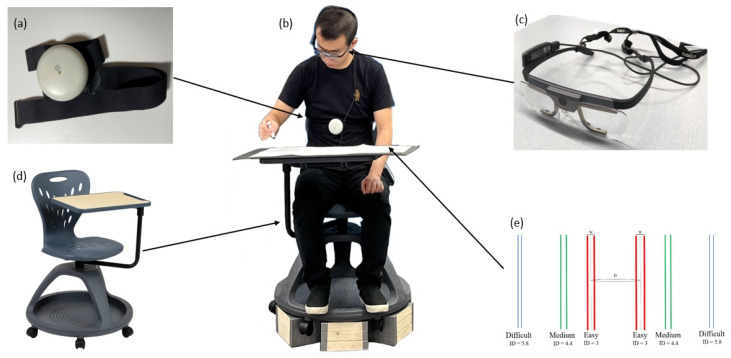
(**a**) Aidlab Aidmed-One physiological monitoring device; (**b**) a researcher carrying out aiming tasks with all the equipment; (**c**) Tobii Pro Eye-tracking Glasses-2; (**d**) rotating chair with a desk; (**e**) aiming task paper with three IDs (Red lines: Easy; Green lines: Medium; Blue lines: Difficult).

The experiment room was lit with fluorescent lighting. The physiological monitoring device (Aidlab Aidmed-One, Aidlab, Gdańsk, Poland) shown in Figure 1a was used to track participants’ HR and RR. The eye-tracking glasses (Tobii Pro Glasses-2 50 Hz, Tobii Technology Inc., Danderyd, Sweden) shown in Figure 1c were used to record videos from participants’ perspectives and to record eye gaze trajectory data and eye metrics. Two video cameras were placed at two different positions to record the experiment process.

### 2.2. Participants

A total of 28 participants were recruited for the experiment. Each participant read the instructions before the experiment. All participants were right-handed and had no prior knowledge of the experiment or training under the in-motion conditions.

### 2.3. Tasks

The participants were asked to perform aiming tasks using provided pens and to mark points within the pairs of target areas labeled on the task papers back and forth repeatedly, as shown in Figure 1. The aiming tasks were conducted under both stationary and in-motion conditions, which will be further elaborated in the next section. The aiming tasks’ IDs were determined by changing the target sizes (W) and distances (D) between the centerlines of the targets based on Fitts’s Law [25] and Shannon’s formulation [26], as shown in Equation (1).

(1) $$\mathrm{ID}=\log_{2}\left(\frac{D}{W}+1\right)$$

Three IDs were set for the aiming tasks, as shown in Figure 1e and Table 1. For each ID, there were two target areas. Each target area was defined by two lines that were close to each other and with identical colors, as shown in Figure 1e.

### 2.4. Experimental Conditions

A rotating chair with a tabletop attached, shown in Figure 1d, was used for both the in-motion and stationary conditions. The task paper (Figure 1e) was placed in front of the participants on the desk attached to the rotating chair. Tasks were conducted on this chair under both stationary and in-motion conditions.

For the experiment under stationary conditions, a researcher firmly held the chair to prevent the chair from moving; participants were asked to place their feet on the ground to further prevent the chair from moving.

For the experiment under in-motion conditions, a researcher rotated the chair about the participant’s vertical axis (yawing motion) clockwise and counterclockwise within a 120-degree angle region with an average rotation speed of ω = 60 degrees/s (1.047 rad/s). The researcher who rotated the chair wore AirPods (Apple Inc., Cupertino, CA, USA) while rotating the chair. The AirPods played tick tock sounds every second so the researcher could follow the sound rhythm to rotate the chair with a consistent speed and rhythm. The chair’s rotation was controlled manually, following specific rhythms, resulting in predictable, quasi-rhythmic, and roughly sinusoidal movements.

### 2.5. Procedures

A researcher provided uniform instructions to all participants before the experiment. Subsequently, each participant was instructed on how to affix the Aidmed-One, shown in Figure 1a, to the xiphoid process and correctly wear the eye-tracking glasses shown in Figure 1c. The participants were comfortably seated in the rotating chair, with a tablet and the task paper positioned at the centerline of their field of vision, as depicted in Figure 1b. Each participant completed the aiming task under six different experimental conditions, including two platform conditions (stationary, in-motion) and three IDs (easy, medium, difficult). To minimize any bias from learning effects, the order of these experimental conditions was counterbalanced. In the stationary conditions, a researcher verbally started the task with the command “Go”. In the in-motion conditions, a researcher initially rotated the chair back and forth for 10 s and then instructed the participants to begin the task with the same command, “Go”. The participants were tasked with swiftly aiming at and marking the points between the target areas while maintaining accuracy within the designated target areas. A researcher quietly kept track of the aiming actions made by each participant. The participants were required to stop the task immediately and prepare for the next experimental condition. After completing the trial under all experimental conditions, each participant was asked to complete a post-trial survey.

### 2.6. Data Acquisition and Data Analysis

Each participant completed reciprocal aiming tasks under the easy, medium, and difficult ID conditions under both the in-motion and stationary conditions. The head-mounted eye-tracking glasses recorded a video of the participant’s task performance from the their perspective. The performance videos were analyzed frame by frame to identify the MT and task errors. Specifically, the time (s) taken to complete the first 30 aiming trials was computed; then, we divided the time by 30 to obtain the mean MT for each aiming trial. The number of aiming trials outside the target areas were counted as the number of errors for each condition.

The eye-tracking glasses continuously recorded participants’ gaze movement trajectories during the tasks. After the experiment, all the eye-tracking metrics were outputted from the iMotions software (Version: 9.4.34558.0). The total number of fixations and saccades were computed during the first 30 aiming trials. Each fixation and saccade had different durations [27], whereas the mean durations of the fixations and saccades were obtained for each experimental condition.

Participants’ heart rate (HR) and respiration rate (RR) were extracted from the Aidmed-One, a wearable device monitoring participants’ physiological response.

To investigate how the in-motion conditions impacted human performance, a 2-platform condition (stationary vs. in-motion) × 3-ID (easy vs. medium vs. difficult) within-subjects ANOVA model was used for statistical analysis, with repeated measures on both factors. The IBM SPSS Statistics (Version: 29.0.1.0) was used for data processing. Results are reported as mean ±95% Confidence Interval unless defined otherwise. *p* < 0.05 was considered indicative of a significant difference.

### 2.7. Post-Trial Survey

At the end of study, each participant was required to complete a 7-question survey to self-assess their task performances. The survey is shown in Appendix A of this paper.

### 2.8. Angular Momentum of Hands and Arms

When participants conducted aiming tasks under in-motion conditions, two effects impacted human performance due to chair motion. The first effect was vestibular stimulation, involving chair rotations that triggered the vestibular system. The second effect resulted from the angular inertia of limbs induced by the chair’s motion. We calculated the average moment of inertia “I” of right limb. Using the moment of inertia “I”, we determined the average angular momentum “L” that participants had to counter under in-motion conditions. Equation (2) illustrates the moment of inertia “I”, where “M” represents the arm mass, and “r” is the distance between the object’s tip and the axis of rotation, which is the arm length.

(2) $$I=\frac{1}{3}\times M\times r^{2}$$

Equation (3) shows the angular momentum “L”, where “I” is the moment of inertia, and “ω” is the angular velocity of the object; ω = 60 degrees/s (1.047 rad/s).

(3) L=I×ω

The average moment of inertia “I” and angular momentum “L” were calculated based on the arm mass “M” [28] and arm length “r” [28]. The calculated results regarding “I” and “L” are presented in Section 3.6.

## 3. Results

Among the 28 participants, 3 participants’ data were neglected due to software and hardware issues. For the remaining 25 participants (12 males and 13 females; mean age: 25.6), each has a pair (one for stationary, one for in-motion) of MT, fixations, saccades, errors, HRs, and RRs for the three different IDs (easy, medium, and difficult). Thus, 75 pairs of data (25 participants with three IDs) were collected for the MT, fixations, saccades, errors, HR, and RR analysis. All 28 participants completed the post-trial survey, and the data were analyzed to determine the results of the survey. We checked the normalization of the data and found that the data distribution approximated a Gaussian distribution. Cell means and marginal means of the abovementioned parameters are displayed in Table 2 and Table 3, respectively.

### 3.1. Movement Time (MT)

The ANOVA model revealed a significant interaction between the task IDs and the platform conditions (F = 36.36, *p* < 0.001) with respect to the MT.

The ANOVA model revealed a significant main effect for platform conditions (F = 55.38, *p* < 0.001), as shown in Table 3. The main effect of platform conditions was consistent with the simple effects observed for each task difficulty, with the overall MT being significantly longer under the in-motion conditions than under the stationary conditions (*p* < 0.001).

There was a significant descriptive main effect for the task IDs (F = 203.14, *p* < 0.001). The main effect of the IDs was also consistent with the simple effects observed for both platform conditions, with the MT being significantly longer for the difficult tasks than for the medium and easy tasks (*p* < 0.001), and significantly longer for the medium tasks than for the easy tasks (*p* < 0.001).

**Figure 2:**
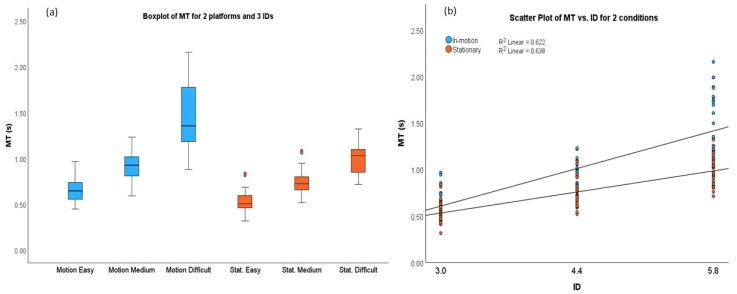
(**a**) Box plots of MT for two platform conditions (blue: in-motion; orange: stationary). (**b**) Scattered plots and linear regressions of MT for two platform conditions.

The results presented in Table 2 and Figure 2a show that the MT was significantly longer under the in-motion conditions than under the stationary conditions for all task IDs (*p* < 0.001). The MT was significantly longer for the difficult tasks than for the medium and easy tasks under both the in-motion and stationary conditions (*p* < 0.001). Moreover, the MT for the medium tasks was significantly longer than that for the easy tasks under both the in-motion and stationary conditions (*p* < 0.001).

The average MT was 33.3% longer under the in-motion conditions (MT__in-motion_ = 1.01 s) than under the stationary conditions (MT__stationary_ = 0.76 s). Specifically, under the in-motion conditions, the average MT was 21.9%, 22.3%, and 47.8% longer for the easy, medium, and difficult tasks, respectively, compared to the average MT values for each task ID under the stationary conditions. According to Fitts’s Law [25], the MT is a linear function of the IDs. Equation (4) expresses the relationship between the MT and IDs.

(4) MT=a+b×ID

where “*a*” and “*b*” are constants that depend on different input methods and experiment settings. The linear relationships between the MT and IDs for the stationary and in-motion conditions are indicated in Figure 2b and represented in Equations (5) and (6), respectively.

(5) $$\mathrm{MT}=0.05+0.16\times \mathrm{ID}\left(3\leq \mathrm{ID}\leq 5.8\right)(R^{2}=0.638)$$

(6) $$\mathrm{MT}=-0.26+0.29\times \mathrm{ID}\left(3\leq \mathrm{ID}\leq 5.8\right)(R^{2}=0.622)$$

Figure 2a shows that the change in the median of the MT during the in-motion conditions became steeper with the increase in the IDs. The constant “b” is larger in Equation (6) than Equation (5) due to various reasons, such as the fact that the in-motion platforms led to a larger initial impulse of movement [29]. This will be further discussed in the Section 4.

### 3.2. Fixations and Saccades

The average durations of each individual eye fixation and saccade were analyzed using the ANOVA model. The ANOVA showed that there was no significant interaction between the task IDs and platform conditions for the fixation duration (F = 1.96, *p* = 0.17), nor for the saccade duration (F = 4.37, *p* = 0.022). However, it revealed significant differences in the fixation and saccade durations for both the platform conditions and the IDs.

**Figure 3:**
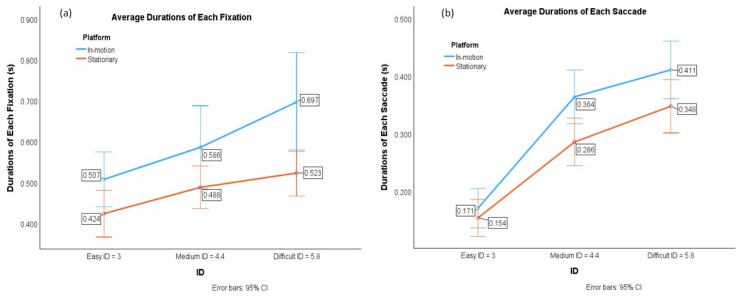
(**a**) Average durations of each eye fixation; (**b**) average durations of each eye saccade.

As shown in Figure 3a,b and Table 3, the results show that both the average durations of each fixation and saccade were significantly longer under the in-motion conditions than they were under the stationary conditions (Fixation__in-motion_ = 0.597 ± 0.081; Fixation__stationary_ = 0.478 ± 0.050) (Saccades__in-motion_ = 0.315 ± 0.032; Saccades__stationary_ = 0.263 ± 0.036). The fixation durations of the difficult tasks were significantly longer than those of the medium and easy tasks, and the fixation durations of the medium tasks were significantly longer than those of the easy tasks (*p* < 0.001).

### 3.3. Errors

The ANOVA model showed a significant interaction effect between the IDs and the platform conditions (F = 30.06, *p* < 0.001) with respect to the errors.

As shown in Table 3, the model revealed a significant main effect of the errors for the platform conditions (F = 82.97, *p* < 0.001). Overall errors were significantly more present for the in-motion conditions compared to the stationary conditions (*p* < 0.001). The pattern of the main effect was consistent with that of the errors’ simple mean effect observed for the easy, medium, and difficult tasks.

The model indicated a significant main effect for the task IDs (F = 34.77, *p* < 0.001). Specifically, there were significantly more errors in the difficult tasks compared to the medium and easy tasks (*p* < 0.001) and in the medium tasks compared to the easy tasks (*p* = 0.007). Notably, this pattern was observed only under the in-motion condition, whereas under the stationary conditions, there was no significant difference in the errors among the task IDs.

**Figure 4:**
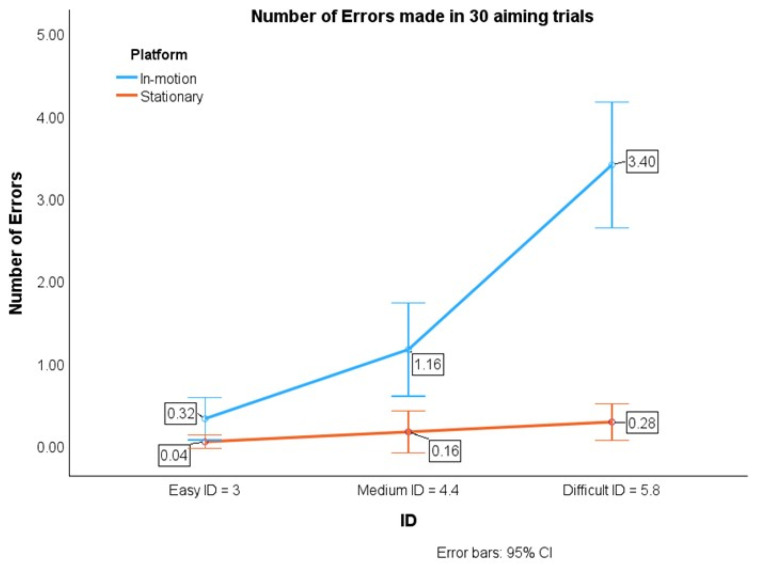
Average error trend lines for two platform conditions.

The errors’ simple means for the easy (*p* = 0.05), medium (*p* = 0.002), and difficult (*p* < 0.001) tasks under the in-motion conditions were significantly higher than those under the stationary conditions, as shown in Table 2 and Figure 4, respectively.

Under the in-motion conditions, the errors’ simple mean for the difficult task was significantly more than that for the medium task (*p* < 0.001) and the easy task (*p* < 0.001). The errors’ simple mean for the medium task was significantly more than that for the easy task (*p* = 0.005). However, there were no significant differences in the errors’ simple mean between the difficult and medium tasks (*p* = 0.185) or between the medium and easy tasks (*p* = 0.376) for the stationary conditions.

The average error for the in-motion conditions (Error__in-motion_ = 1.63) were 10.19 times more than that for the stationary conditions (Error__stationary_ = 0.16). Specifically, for the in-motion conditions, the average error was 8, 7.25, and 12.14 times more than that the average error values for the easy, medium, and difficult tasks, respectively, under stationary conditions. Overall, the error increased with the increase in IDs.

Importantly, the relationship between the IDs and errors was typically linear under stationary conditions only. However, under the in-motion conditions, the errors did not follow a linear pattern with the IDs, as shown in Figure 4.

### 3.4. Respiration Rate (RR)

The ANOVA model showed no significant interaction between the task IDs and platform conditions (F = 0.92, *p* = 0.40) for the RR. However, it revealed significant differences between the in-motion and stationary conditions for the RR (F = 8.08, *p* = 0.009).

As shown in Table 2, the RR simple mean for the easy task under the in-motion conditions was significantly higher than that under the stationary conditions (*p* = 0.013). The RR simple mean for the medium task under the in-motion conditions was significantly higher than that under the stationary conditions (*p* = 0.037). However, there was no significant difference in the RR simple mean for the difficult task between the in-motion and stationary conditions (*p* = 0.180).

Moreover, under the in-motion conditions, there were no significant differences in RR simple mean between the difficult and medium tasks (*p* = 0.237), between the difficult and easy tasks (*p* = 0.097), or between the medium and easy tasks (*p* = 0.706). However, under the stationary conditions, the RR simple mean for the difficult task was significantly higher than that for the easy task (*p* = 0.003), but no significant differences between the difficult and medium tasks (*p* = 0.153) or the between medium and easy tasks (*p* = 0.192) were found.

### 3.5. Heart Rate (HR)

There was no significant interaction between the task IDs and the platform conditions (F = 1.26, *p* = 0.29) for the HR. However, we found significant differences between the in-motion and stationary conditions for the HR (F = 4.42, *p* = 0.046).

As shown in Table 2, only the HR simple mean for the medium task under the in-motion conditions was significantly higher than that under the stationary conditions (*p* = 0.042). However, there was no significant difference in HR simple mean for the easy task (*p* = 0.113) and difficult task (*p* = 0.746) between the in-motion and stationary conditions.

Under the in-motion conditions, there were no significant differences in the HR simple mean between the difficult and medium tasks (*p* = 0.627), between the difficult and easy tasks (*p* = 0.922), or between the medium and easy tasks (*p* = 0.648). However, under the stationary conditions, the HR simple mean for the difficult task was significantly higher than that for the easy task (*p* = 0.013), but no significant differences between the difficult and medium tasks (*p* = 0.054) or between the medium and easy tasks (*p* = 0.288) were found.

### 3.6. Moment of Inertia and Angular Momentum

Table 4 shows the average arm mass “M” [28], arm length “r” [28], and moment of inertia “I” calculated from Equation (2) based on “M” and “r”, as well as the angular momentum “L” calculated from Equation (3) based on “M” and “r”.

### 3.7. Post-Trial Survey

Most of the participants reported they focused on movement accuracy more than speed when performing tasks for both stationary (24 out of 28) and in-motion (28 out of 28) conditions in the Q1 and Q2. However, there were still more errors made on the in-motion (1.63 errors) than the stationary conditions (0.16 errors). In terms of content validity in the Q3, most participants (24 out of 28) agreed the experiment simulated and replicated the real-world in-motion situations. All the participants agreed the tasks were more challenging on the in-motion than stationary conditions in the Q4. Some participants (18 out of 28) thought the reason was due to visual related problems in the Q5, while most (27 out of 28) thought to be non-visual problems in the Q6. According to the Q7, there was no apparent predisposition on whether the in-motion conditions would cause more mental than physical demand. Whereas all participants reported more efforts were put on the tasks along with undergoing greater cognitive load on in-motion conditions.

## 4. Discussion

Our hypotheses were supported by the results of our experiment. We found that participants completed aiming tasks with prolonged MT and increased errors for the in-motion conditions compared to the original Fitts’s Law model. The participants’ raised HR and RR were recorded for the in-motion conditions. The average durations of each eye fixation and saccade were obviously longer under the in-motion conditions than under the stationary conditions. It is worth noting that the participants spent significantly longer times on both fixations and saccades under the in-motion conditions than under the stationary conditions. Therefore, the in-motion conditions demanded more effort from the participants in terms of processing visual information (longer fixation durations), and the participants took longer to locate the target (longer saccade durations) [27,30]. Based on the experimental results described in Section 3, some possible reasons behind the prolonged MT, longer durations of fixations and saccades, and greater amount of errors under the in-motion conditions are proposed in this section.

Head movement, the relative positional change between target areas and eyes, and the disturbances to their vestibulo-ocular reflexes (VORs) [31,32] under the in-motion conditions impacted the subjects’ visual processing and performances. The ocular pursuit [33,34,35,36] performance was optimal for target speeds ranging between 15°/s and 30°/s [37,38], which was less than the platform rotation speed (ω = 60°/s). Therefore, during the in-motion conditions, both ocular pursuit and VOR disturbance happened simultaneously. The in-motion conditions caused visual instability and disturbed subjects’ VORs [39,40,41,42]. Furthermore, the in-motion conditions stimulated and excited the human vestibular system, leading to irregular VORs [43,44,45]. Consequently, the participants’ MT values increased, with longer durations of saccades and fixations being needed to allow them to aim at the target areas before the visual information was perceived and processed under the in-motion conditions due to delayed visual processing progress and unstable VORs, consequently leading to more errors.

Second, the participants were in a non-inertial frame under the in-motion conditions, leading to the inconsistent muscular force application on their upper limbs, which adversely deteriorates the aiming motor skills. As listed in Table 4, at least an extra 0.340 $\frac{\mathrm{kg}\cdot m^{2}}{s}$ of angular momentum was applied to the participants’ right hands and arms. The change in angular momentum (ΔL) is shown in Equation (7), where “τ” is the average net torque, “Δt” is the time interval that torque was applied for, “r” is the length of the lever perpendicular to the net external force “F_⊥_”, and “F_⊥_” is the net external force perpendicular to the arm.

(7) $$\Delta L=\tau *\Delta t=r*F_{⊥}*\Delta t$$

When extra angular momentum was applied, the average movement time Δt and the net external force F_⊥_ would accordingly increase to compensate the extra angular momentums caused by the rotation. Specifically, the subjects tended to apply more muscular force to initiate the movement [46,47] and consequently terminate the action under the in-motion conditions. Therefore, the inconsistent muscle force deteriorated the aiming motor skills. This effect was more obvious when the extra angular momentum accumulated, especially in the high-ID tasks. Thus, the prolonged MT and errors were more pronounced in the difficult tasks compared to the easy and medium tasks. Participants’ self-assessments at the end of the experiment, as shown in Appendix A, also supported our arguments, as in Q6, some participants reported that the difficulty they experienced in completing the tasks was not mainly caused by the visual problems.

**Figure 5:**
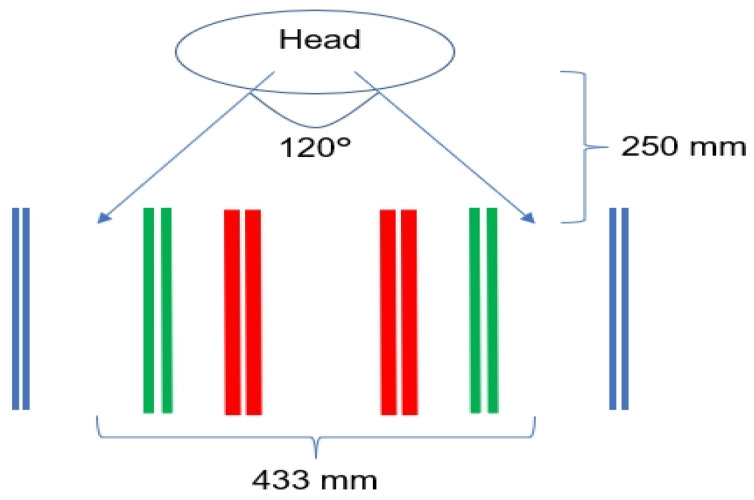
Relationships between eyes and the task paper (Red lines: Easy; Green lines: Medium; Blue lines: Difficult).

Third, the physical constraint of the subjects’ bodies under the in-motion conditions worsened their performances. Human eye vision can span approximately 120 degrees of arc [48]. In our experiment, participants’ head positions were approximately 250 mm from the task paper, providing an eye vision field coverage of about 433 mm on the task paper, as represented in Figure 5.

The covered distance accommodated the easy (centerline distance: 140 mm) and the medium tasks (centerline distance: 301.5 mm) but was less ideal for the difficult task (centerline distance: 547.2 mm). The reduced eye vision field provoked additional challenges during the difficult tasks, where the target areas were beyond the ordinary human visual field and required the participants to move their head. Under the in-motion conditions, head movements were more difficult to initiate and synchronize with the movement of their eyes. Thus, the participants spent more time on eye saccades to locate the target, and more time on eye fixations to lock the target. As a result, the largest MT increments between the in-motion and stationary conditions were observed in the difficult tasks (difficult: 47.8%) compared to the other two tasks (medium: 22.3%; easy: 21.9%).

Lastly, the stimulation of the vestibular system may have adversely affected aiming task performance under the in-motion conditions. When the human body is under the in-motion conditions, the hair cells in the semicircular canals detect the repetitively abnormal head rotations around the vertical axis [49], which may cause vertigo and inconsistencies in information intake between the vestibular and visual pathways. Therefore, aiming performance may deteriorate temporarily, explaining the prolonged processing times and longer MTs under the in-motion conditions.

## 5. Conclusions

The in-motion conditions significantly affected human aiming motor skills, as evidenced by the prolonged MTs (with prolonged durations of both eye fixations and saccades) and greater amount of performance errors. These effects were more pronounced for the higher-ID tasks under the in-motion conditions. The MT-ID and Error-ID relationships for the in-motion conditions have steeper slopes than those for the stationary conditions. Possible reasons for this include differences visual processing progress; disturbed VORs; altered muscular force generation due to inertia; kinesiological and physical differentiations due to inertia; the various eye vision angles; and the stimulation of vestibular systems. In addition to prolonging MTs, prolonging the durations of fixations and saccades, and generating more errors, the in-motion conditions also resulted in significantly higher HR and RR values.

Further research on these challenges under in-motion scenarios will help improve in-motion system design and enhance human aiming performance in various settings. This research will also help to create specific training protocols to mitigate the negative effects of in-motion platforms. Ultimately, it will allow us to take precautions against operational errors, maximize workability, and minimize physical and cognitive loads when working under in-motion conditions.

## 6. Limitations

This study has some limitations. Firstly, our experimental setup featured a fixed rhythm in the rotating motion, allowing participants to predict and develop strategies to cope with this pattern. In future experiments, we intend to introduce unpredictable moving platforms to investigate the differences in motor skills under predictable and unpredictable motion conditions. Secondly, in the present study’s experiment, the participants had to adapt to and manage both motion inertia and vestibular stimulation generated by the rotating chair under the in-motion conditions. To isolate and study these two factors separately, it would be reasonable to conduct experiments assessing motor skills exclusively under the influence of vestibular stimulation. Thirdly, there is room for improvement in assessing physical and mental stress. We plan to include additional data channels, such as pupil diameters, saccadic eye patterns, and blink rate, to enhance the sensitivity in capturing changes in participants’ physical and mental states during task performance.

Looking forward, we propose two additional research directions for this study. To examine how individuals adapt to the vestibular feedback in their motor skills over time, we plan to have participants perform tasks repeatedly under in-motion conditions during an extended training phase. Additionally, participants will be subjected to auditory interferences to investigate their adaptation to and management of both auditory and vestibular feedback while preserving motor skills. This is critical, as it simulates a common scenario for aircraft pilots who must multitask while maintaining motor skills. Lastly, we will investigate the effects on human motor performance under more complicated and hybrid motion conditions using flight simulators. Thus, more real-world scenarios will be simulated and investigated.

## Figures and Tables

**Table 1:** Relationships of IDs with width of target and centerline distance of targets.

	Easy (ID = 3)	Medium (ID = 4.4)	Difficult (ID = 5.8)
Width (mm):	20	15	10
Centerline Dist. (mm):	140	301.5	547.2

**Table 2:** Cell means ± 95% Confidence Interval with respect to two platform conditions and three IDs.

		Easy	Medium	Difficult
MT (s)	In-motion	0.65 ± 0.058	0.92 ± 0.071	1.46 ± 0.156
	Stationary	0.54 ± 0.049	0.75 ± 0.059	0.99 ± 0.066
	*p* value	<0.001	<0.001	<0.001
Errors	In-motion	0.32 ± 0.26	1.16 ± 0.57	3.40 ± 0.76
	Stationary	0.04 ± 0.08	0.16 ± 0.26	0.28 ± 0.22
	*p* value	=0.05	=0.002	<0.001
Fixation (s)	In-motion	0.507 ± 0.067	0.586 ± 0.102	0.697 ± 0.121
	Stationary	0.424 ± 0.057	0.488 ± 0.052	0.523 ± 0.057
	*p* value	=0.008	=0.049	=0.009
Saccades (s)	In-motion	0.171 ± 0.034	0.364 ± 0.046	0.411 ± 0.050
	Stationary	0.154 ± 0.032	0.286 ± 0.041	0.348 ± 0.046
	*p* value	=0.24	=0.001	=0.006
RR (bpm)	In-motion	15.7 3 ± 2.03	16.12 ± 2.08	17.13 ± 1.66
	Stationary	13.43 ± 2.58	14.80 ± 2.12	16.00 ± 2.30
	*p* value	=0.013	=0.037	=0.180
HR (bpm)	In-motion	86.48 ± 6.35	87.21 ± 6.78	86.63 ± 6.27
	Stationary	84.05 ± 5.52	85.07 ± 6.01	86.35 ± 5.93
	*p* value	=0.113	=0.042	=0.746

**Table 3:** Marginal means ± 95% Confidence Interval with respect to two platform conditions and three IDs.

	MT (s)	Errors	Fixation (s)	Saccades (s)	RR (bpm)	HR (bpm)
In-motion	1.011 ± 0.085	1.63 ± 0.35	0.597 ± 0.081	0.315 ± 0.032	16.33 ± 1.61	86.78 ± 6.25
Stationary	0.758 ± 0.053	0.16 ± 0.15	0.478 ± 0.050	0.263 ± 0.036	14.74 ± 2.10	85.16 ± 5.74
*p* value (platform)	<0.001	<0.001	=0.005	=0.001	=0.009	=0.046
Easy	0.595 ± 0.047	0.18 ± 0.13	0.466 ± 0.055	0.163 ± 0.030	14.58 ± 2.14	85.27 ± 5.75
Medium	0.832 ± 0.057	0.66 ± 0.33	0.537 ± 0.064	0.325 ± 0.038	15.46 ± 2.00	86.14 ± 6.32
Difficult	1.226 ± 0.101	1.84 ± 0.43	0.610 ± 0.070	0.379 ± 0.043	16.56 ± 1.82	86.49 ± 6.04
*p* value (ID)	<0.001	<0.001	<0.001	<0.001	=0.046	=0.36
*p* value (interaction)	<0.001	<0.001	=0.17	=0.022	=0.4	=0.29

**Table 4:** Average moment of inertia and angular momentum.

	“M” (kg) [28]	“r” (m) [28]	“I” ($kg\cdot m^{2}$)	“L” ($kg\cdot m^{2}\cdot s^{-1}$)
Males	4.678	0.593	0.548	0.574
Females	3.048	0.566	0.325	0.340

## Data Availability

Data are available from the corresponding author upon reasonable request.

## Appendix A

**Table A1:** Post-trail survey.

1. I focused on movement accuracy more than speed when performing the aiming tasks in the stationary condition.				
Strongly Agree	Agree	Neutral	Disagree	Strongly Disagree
12	8	4	4	0
2. I focused on movement accuracy more than speed when performing the aiming tasks in the rotating condition.				
Strongly Agree	Agree	Neutral	Disagree	Strongly Disagree
14	12	2	0	0
3. Performing the aiming tasks in the rotating condition is similar to what I have experienced in moving vehicles, aircrafts, and/or ships.				
Strongly Agree	Agree	Neutral	Disagree	Strongly Disagree
4	11	9	4	0
4. Performing the aiming tasks in the rotating condition is more challenging than in the stationary condition.				
Strongly Agree	Agree	Neutral	Disagree	Strongly Disagree
25	3	0	0	0
5. Performing the aiming tasks in the rotating condition is more challenging due to visual related problems, such as finding the targets, and maintaining visual focus on the task papers.				
Strongly Agree	Agree	Neutral	Disagree	Strongly Disagree
4	9	5	9	1
6. Performing the aiming tasks in the rotating condition is more challenging due to non-visual problems, such as maintaining hand stability, and dealing with dizziness and nausea.				
Strongly Agree	Agree	Neutral	Disagree	Strongly Disagree
11	13	3	1	0
7. Performing the aiming tasks in the rotating condition is more demanding on my mental capacities (attention, accuracy) than physical capacities (strength, speed).				
Strongly Agree	Agree	Neutral	Disagree	Strongly Disagree
7	6	7	5	3
